# Rectal Bezoar: A Rare Cause of Intestinal Obstruction

**DOI:** 10.7759/cureus.35726

**Published:** 2023-03-03

**Authors:** Fábio Caleça Emidio, Rafaela C Pereira, Rosário Blanco Saez, Teresa Abegão, Ana S Ribeiro

**Affiliations:** 1 Department of Internal Medicine, Centro Hospitalar Universitário do Algarve, Hospital de Faro, Faro, PRT

**Keywords:** chatgpt, rectal bezoar, rectal diseases, endoscopy, intestinal obstruction, bezoars, adult

## Abstract

Bezoars are conglomerates of undigested contents that accumulate in the gastrointestinal tract. They can have different compositions, such as fibers, seeds, vegetables (phytobezoars), hair (trichobezoars), and medication (pharmacobezoars). Bezoars are typically caused by an impaired grinding mechanism of the stomach or interdigestive migrating motor complex, but the composition of ingested material can also play a role in their formation. Gastric dysmotility, previous gastric surgery, and gastroparesis are some of the risk factors that can increase the likelihood of developing bezoars. While bezoars are usually asymptomatic and found in the stomach, they can sometimes migrate to the small intestine or colon and cause complications such as intestinal obstruction or perforation. Endoscopy is essential for diagnosis and etiology, and treatment depends on the composition, which can include chemical dissolution or surgical intervention. We present a case of an 86-year-old woman, who had a bezoar located in an unusual location (rectum), most likely due to migration. This condition led to symptoms of intermittent intestinal obstruction and rectal bleeding. However, due to anal stenosis, the patient was unable to expel the bezoar. Its removal was not possible through various endoscopic techniques. Therefore, it was removed via fragmentation, using an anoscope and forceps, due to its hard/stone-like consistency. This case highlights the importance of considering bezoars in the differential diagnosis of gastrointestinal bleeding and illustrates the importance of prompt diagnosis and appropriate techniques for the removal of bezoars.

## Introduction

Bezoars are conglomerates of undigested material that accumulate in the gastrointestinal tract. They are formed from a variety of substances that may have been intentionally or unintentionally ingested. Therefore, bezoars may present various compositions, with the most common being made up of fibers, seeds, vegetables (phytobezoar), hair (trichobezoar), medication (pharmacobezoar), and milk protein in infants fed with milk (known as lactobezoars) [1]. The impaired grinding mechanism of the stomach and interdigestive migrating motor complex can cause the formation of gastric bezoars when indigestible material is ingested [2]. While it was once believed that delayed gastric emptying was the sole cause of all bezoars, studies have revealed that many patients with normal or accelerated gastric emptying can also develop them. This suggests that the composition of the ingested material is a critical factor in the pathogenesis. There is also a hypothesis that proline-rich proteins, released by the parotid and submandibular glands, have a strong tendency to bind with tannins, which could promote the formation of bezoars [3]. After formation, bezoars continue to grow due to the ingestion of food containing cellulose and other indigestible materials, which become entangled with protein, mucus, and pectin. Gastric bezoars are infrequent, with an estimated incidence rate of 0.3% observed during upper endoscopy [4]. Mihai et al. [5] reported only 49 cases during a 20-year period in 2013, consisting of 0.068% of all endoscopies. Several risk factors have been identified in individuals with gastric bezoars. One of these is gastric dysmotility, which may result from an underlying anatomic abnormality and increase the likelihood of developing bezoars. Research has revealed that a significant proportion of patients with gastric bezoars have previously undergone gastric surgery (70-94%) and vagotomy with pyloroplasty (54-80%) [6-8]. Patients with gastroparesis are also at a greater risk of developing bezoars due to the stomach's impaired grinding mechanism and interdigestive migrating motor complex. Other factors that may elevate the risk of bezoar formation, particularly in patients with pharmacobezoars, include gastric outlet obstruction, dehydration, the use of anticholinergic agents or opiates, and the use of medications with an insoluble carrying vehicle (such as enteric-coated aspirin and nifedipine) [9,10]. Although generally asymptomatic and mostly found in the stomach, bezoars sometimes migrate to the small intestine or colon, where they can rarely cause acute abdomen with intestinal obstruction or perforation [11]. Endoscopy is essential for establishing diagnosis and etiology. The treatment is guided by its composition and includes chemical dissolution or surgical intervention [12].

## Case presentation

**Figure 1:**
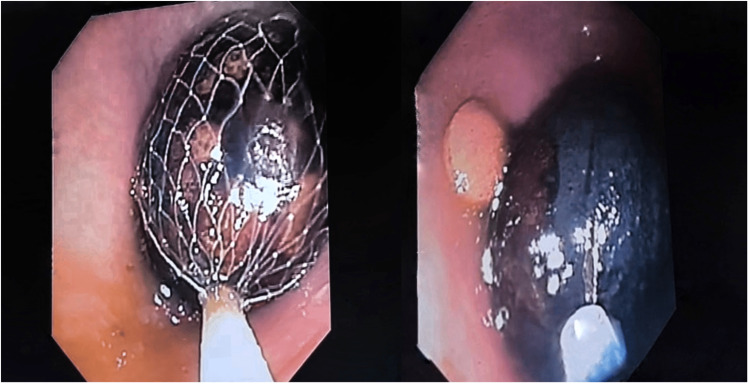
Rectal bezoar: a spherical mass with adjacent undigested pill Spherical bezoar measuring approximately 30 mm in diameter, located in the rectum. Note the coexistence of an adjacent undigested pill.

We present a case of an 86-year-old woman, previously independent, admitted to the department of internal medicine for ischemic stroke. Upon admission, she had a history of constipation for more than three days, which was treated with laxatives, without improvement. On physical examination, the patient presented with a distended and tympanitic abdomen, although with no pain at palpation. After five days, a rectal tube was inserted, with the passage of gas and liquid feces. This was followed by rectal bleeding without an apparent cause and hence colonoscopy was performed. During the examination, a spherical, smooth-surfaced bezoar was visualized at the rectum, measuring 30 mm in diameter, and with a hard/stone-like consistency (Figure 1).

**Figure 2:**
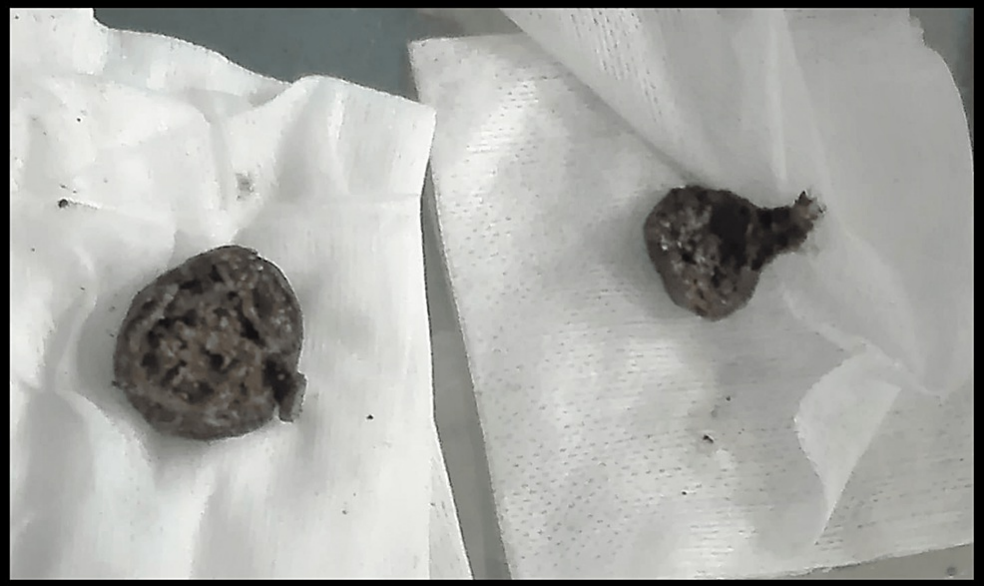
Fragmentation of gastrointestinal bezoar during extraction The gastrointestinal bezoar was fragmented during the extraction process using Magil and Kocher forceps.

Although various endoscopic techniques (polypectomy snare, Dormia basket, net snare, and lithotripter) were attempted, the bezoar could not be removed. Therefore, it was fragmented with the assistance of an anoscope, Magill forceps, and Kocher forceps to facilitate its removal (Figure 2).

## Discussion

Bezoars, as described, are a rare occurrence with an estimated incidence rate of 0.3%, observed during upper endoscopy [4]. However, they can cause serious complications such as intestinal obstruction or perforation, which was the case in our patient. This highlights the importance of prompt diagnosis of bezoars. The presentation of rectal bleeding highlights the importance of considering bezoars in the differential diagnosis of gastrointestinal bleeding. While gastrointestinal bezoars are mostly found in the stomach, they can sometimes migrate to the small intestine or colon, where they can cause acute abdomen with intestinal obstruction or perforation [11]. Treatment depends on their composition and may involve chemical dissolution or surgical intervention [12]. The bezoar in this case could not be removed using various endoscopic techniques, and thus, it was fragmented and removed with the assistance of an anoscope and forceps. This highlights the importance of using appropriate techniques, sometimes atypical ones, for the removal of these conglomerates. The present case also reveals several risk factors that may increase the likelihood of developing this problem. The patient had a history of constipation and ischemic stroke, which may have contributed to gastric dysmotility and impaired grinding mechanism of the stomach, leading to the formation of the bezoar. In addition, the patient was elderly, and age-related changes in the digestive system may have also played a role [13].

## Conclusions

This case describes the presence of a bezoar in an atypical anatomical location (rectum), probably due to migration, associated with clinical symptoms of intermittent intestinal obstruction and rectal bleeding. The bezoar was unable to be expelled due to the coexistence of anal stenosis. Bezoars are a rare occurrence, but they can cause serious complications if not promptly diagnosed and appropriately treated. Understanding the risk factors and appropriate management techniques is crucial in ensuring positive patient outcomes.

## Figures and Tables

**Figure 3:**
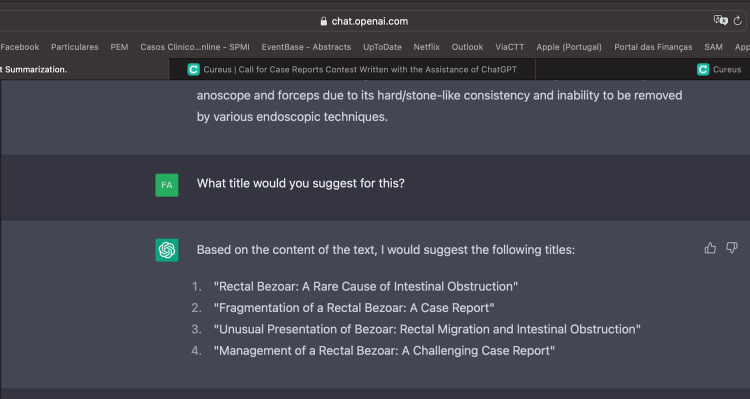
ChatGPT screenshot: suggested title

**Figure 4:**
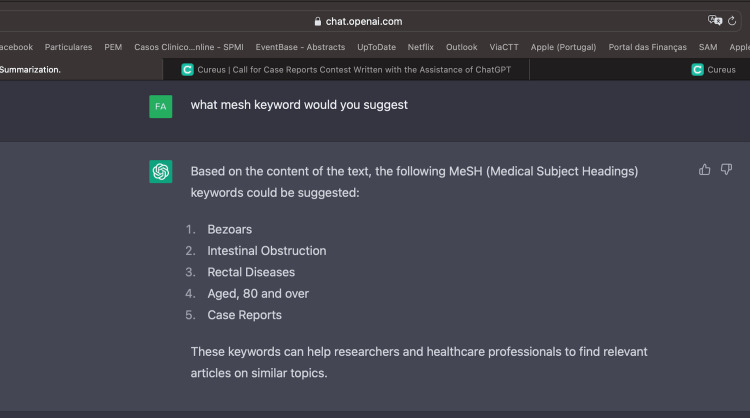
ChatGPT screenshot: suggested keywords

**Figure 5:**
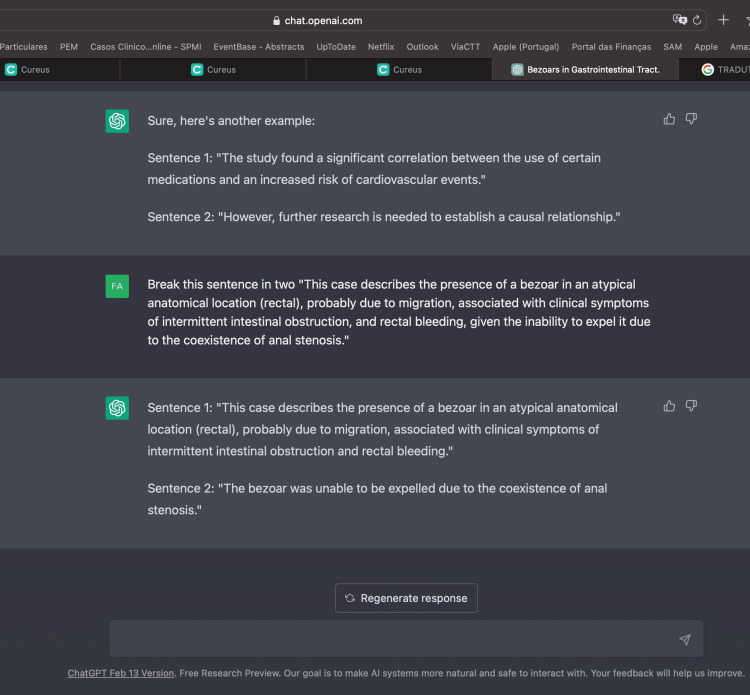
ChatGPT screenshot: suggested conclusion improvement

## Appendices

Parts of this article were improved using "ChatGPT", as stated in the following screenshots (Figures 3-5).
